# Distant Lung Recurrence of Rectal Cancer 20 Years After Primary Surgery

**DOI:** 10.7759/cureus.34122

**Published:** 2023-01-23

**Authors:** Sreekanthan Gobishangar, Sivakumaran Gobinath, Antony J Thanenthiran, Subramaniyam Bakeerathan

**Affiliations:** 1 General Surgery, Faculty of Medicine, University of Jaffna, Jaffna, LKA; 2 General Surgery, Jaffna Teaching Hospital, Jaffna, LKA; 3 Surgical Oncology, Jaffna Teaching Hospital, Jaffna, LKA

**Keywords:** thoracotomy, hemoptysis, adenocarcinoma, lung metastasis, rectal cancer

## Abstract

A male patient in his 70s, who had undergone an abdominoperineal (A1) resection for rectal cancer 20 years prior, presented with hemoptysis. Imaging studies revealed distant lung recurrence, with no evidence of local recurrence. Biopsy revealed an adenocarcinoma, possibly of rectal origin. Immunohistochemical markers were suggestive of rectal cancer metastasis. However, carcinoembryonic antigen (CEA) levels were normal, and colonoscopy did not reveal any metachronous lesions. Curative left upper lobe resection was performed via posterolateral thoracotomy. The patient’s recovery was uneventful.

## Introduction

Colorectal cancer is the third most common cancer in the world and the second most common cancer-causing death as per the GLOBOCAN 2020 [1]. Depending on its stage, it is treated with surgical resection and chemoradiation. Rectal cancer can present with distant metastasis, especially to the lungs and liver, and may require metastasectomy [2]. The time interval between the colonic resection and the metastasectomy is termed the disease-free interval (DFI). As defined by the National Cancer Institute (NCI) at the National Institutes of Health (NIH), distant recurrence is when cancer has recurred in another part of the body far from where the original tumor formed first. Cases of distant lung recurrence 20 years after curative surgery for rectal cancer, without local recurrence, are rare. This DFI is beyond the limits described in surveillance guidelines [3]. This report presents a rare case of such a presentation.

## Case presentation

A male patient in his 70s was diagnosed with rectal cancer and underwent abdominoperineal resection 20 years prior. He initially presented with painless rectal bleeding, which was investigated using facilities available at that time. Colonoscopy revealed a malignant lesion located 5 cm from the anal verge. No other synchronous lesions were observed. Histological findings were suggestive of an infiltrative, moderately differentiated adenocarcinoma with many villous structures, and grade 3 dysplasia. Chest and liver imaging did not reveal any metastatic lesions. Abdominoperineal resection was performed, and a biopsy revealed complete excision of the tumor with adequate margins. The patient subsequently underwent six cycles of adjuvant chemotherapy with folinic acid, fluorouracil, and oxaliplatin (FOLFOX). He was managed in accordance with the National Comprehensive Cancer Network (NCCN) guidelines, and was followed up with regular imaging, assessment of CEA levels, and colonoscopy for up to five years.

After 20 years, the patient presented with a one-week history of hemoptysis. He was examined for possible causes of hemoptysis at the respiratory unit. He had a dry cough, pleuritic chest pain, no fever, no history of contact with a patient with tuberculosis, no weight loss, and no loss of appetite. He had no history of cigarette smoking or alcohol consumption but had occupational exposure to coal smoke. The patient had no family history of lung or rectal cancer. Physical examination did not elicit icterus, cervical lymphadenopathy, or lung signs.

**Figure 1:**
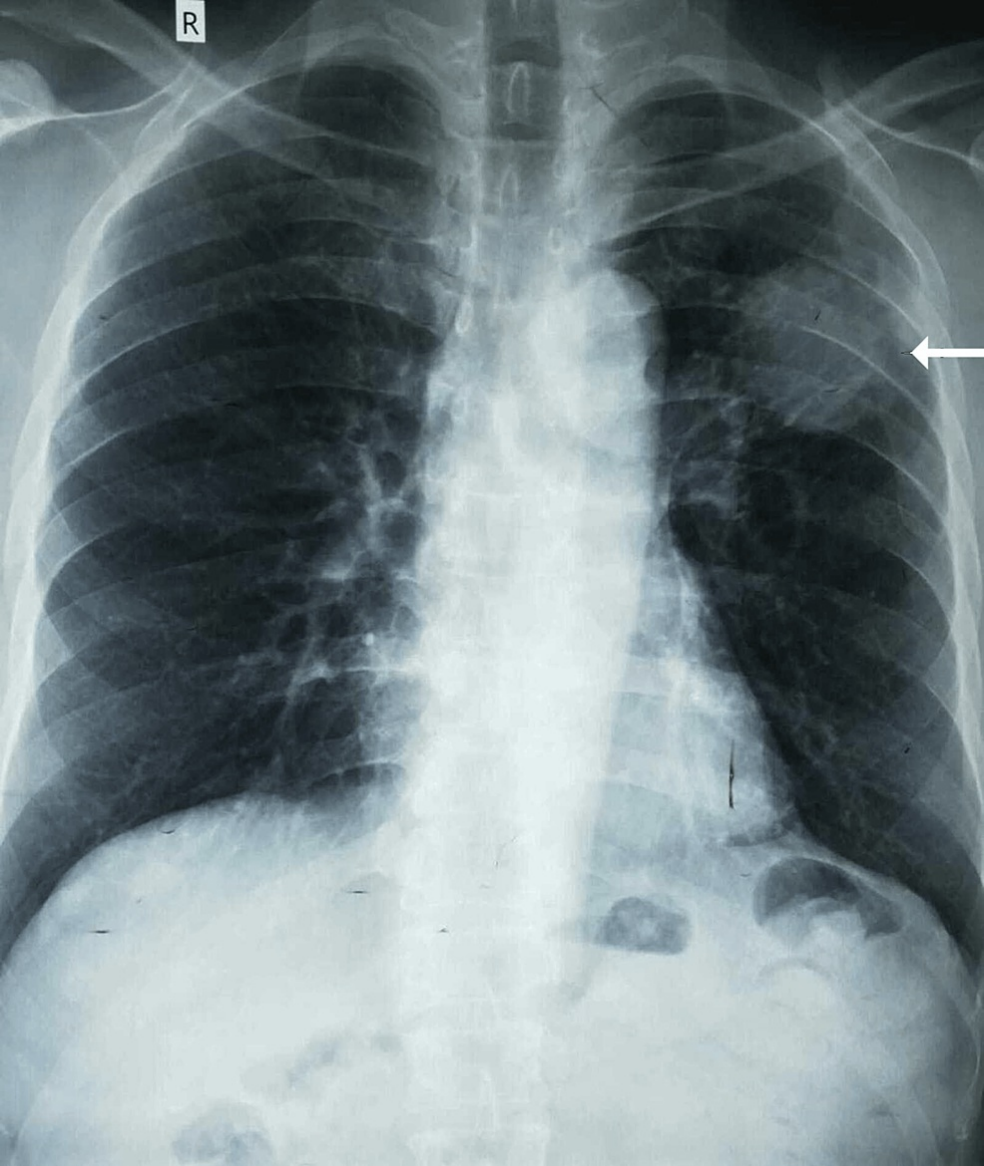
Chest radiographic image showing a mass lesion in the upper lobe of the left lung (white arrow).

**Figure 2:**
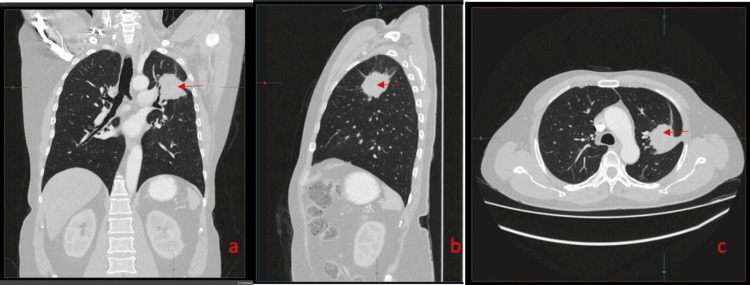
CECT images of the lung showing the tumour in (a) coronal section, (b) sagittal section, and (c) transverse section (red arrows). CECT: contrast-enhanced computed tomography

**Figure 3:**
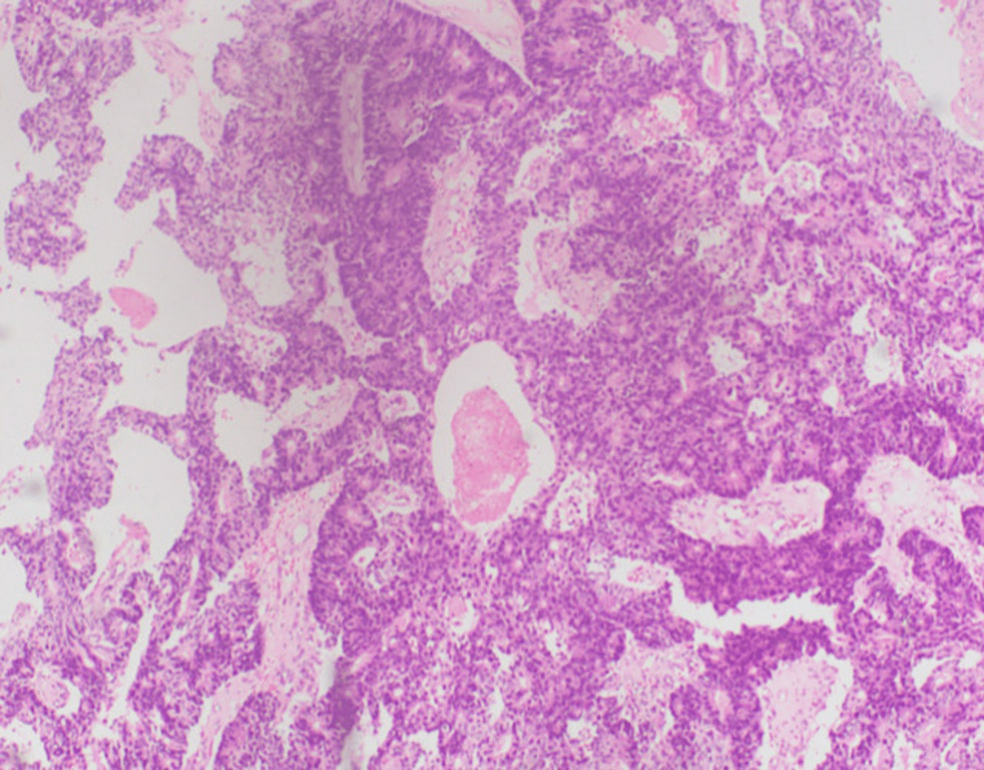
Hematoxylin and eosin stain of the bronchial tissue, original magnification x 100

**Figure 4:**
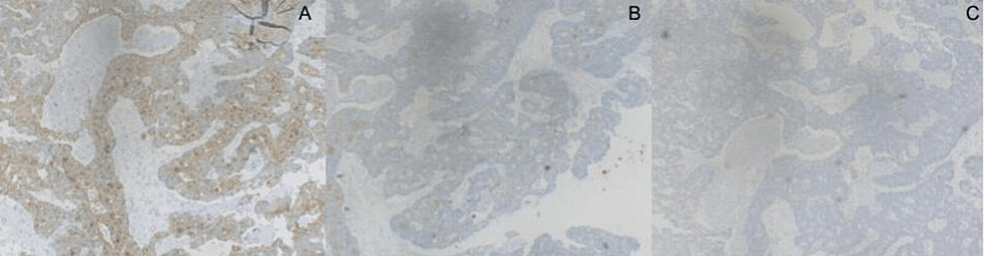
Immunohistochemical observation of the bronchial tissue. (A) CK20 positive, (B) CK7 negative, (C) TTF-1 negative CK20: cytokeratin 20; CK7: cytokeratin 7; TTF-1: thyroid transcription factor

**Figure 5:**
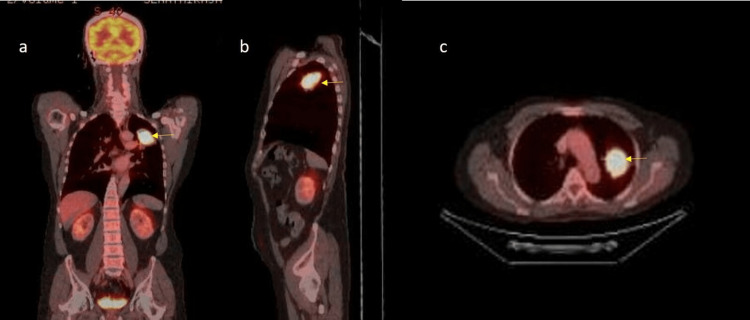
PET images of the lung showing the tumour in (a) coronal section, b) sagittal section, and (c) transverse section (yellow arrows) PET: positron emission tomography

An initial chest radiograph revealed a lesion in the upper lobe of the left lung (Figure 1). CEA was within the normal range. Contrast-enhanced computed tomography (CECT) of the chest, abdomen, and pelvis was performed. CECT showed a single mass in the left upper lobe with contrast enhancement and an irregular outline, measuring 3.4×3.5 cm with involvement of the adjacent pleura along the left lateral chest wall (Figure 2). However, the rest of the lung fields were normal. Bronchoscopy and biopsy were also performed. A contrast-enhancing area in CECT in the deep pelvis at the site of the rectum was seen but was most likely chronic scar tissue at the site of the primary surgery. Biopsy revealed an adenocarcinoma composed of closely packed confluent malignant glands with necrotic areas. The glands were lined by moderately pleomorphic cells with hyperchromatic nuclei and eosinophilic cytoplasm. The morphology was compatible with that of metastatic rectal adenocarcinomas (Figure 3). Immunohistochemistry of the bronchial tissue showed strong positivity for cytokeratin 20 (CK20) and negativity for cytokeratin 7 (CK7) and thyroid transcription factor (TTF-1) (Figure 4). Whole-body positron emission tomography (PET) was performed to exclude any other possible metastatic deposits, while we made plans for curative surgery. A strongly fluorodeoxyglucose (FDG) -avid lesion was identified in the inferior part of the apicoposterior segment of the left upper lobe of the lung, measuring 4.92×4.16×4.14 cm (Figure 5). There was neither mediastinal nor hilar lymphadenopathy, nor pleural or pericardial effusion.

**Figure 6:**
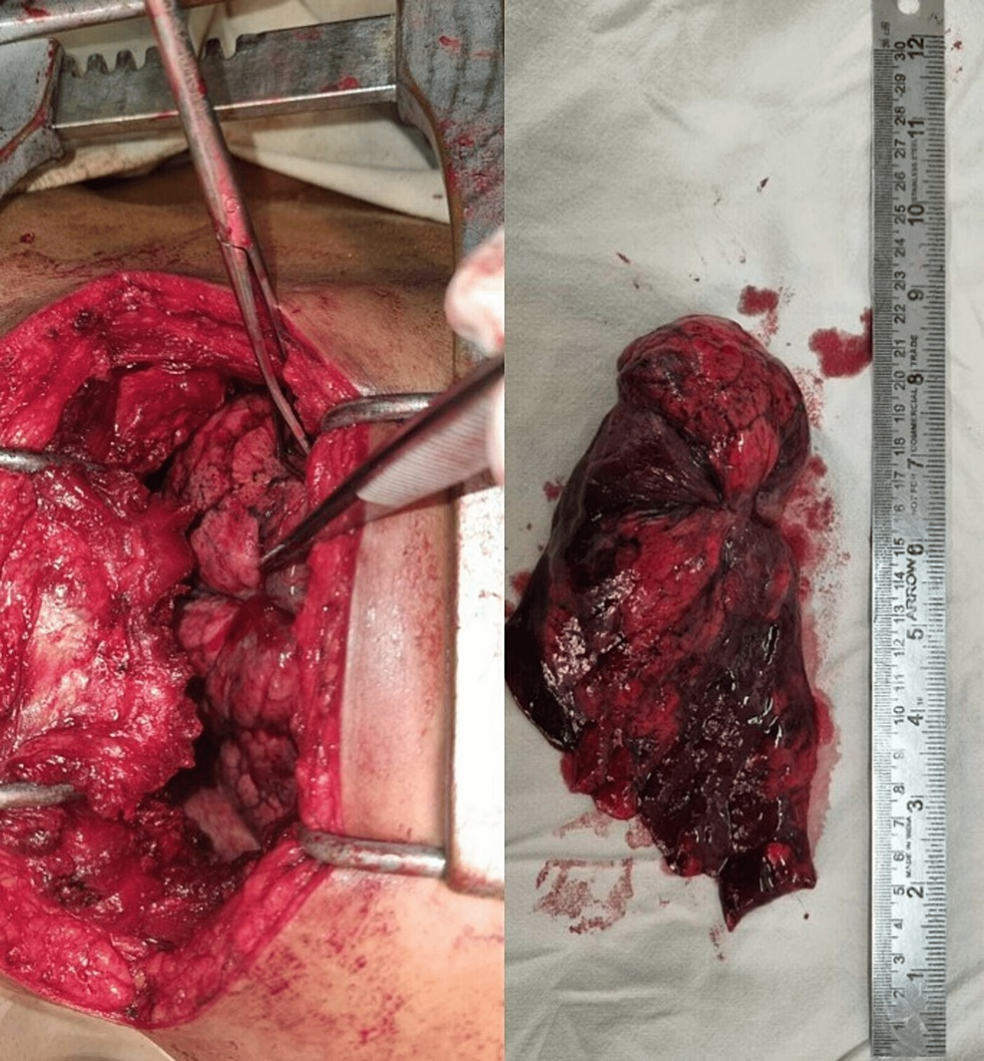
Intraoperative image and the resected specimen of the lung tumour

The case was discussed in a multidisciplinary team meeting and two options were suggested. The first was metastasectomy and adjuvant chemotherapy, while the second was neoadjuvant chemotherapy followed by surgery. The options were discussed with the patient, and he chose the first option. A left upper lobectomy was planned using an open surgical approach. Fitness for surgery was assessed preoperatively. A posterolateral thoracotomy was performed along the fifth intercostal space. Pleural reflections and the lungs were identified, after which the pleural reflections were divided. The upper lobes of the pulmonary artery, bronchus, and pulmonary veins were identified and ligated. The anterior and posterior ends of the oblique fissure were divided using a linear stapler. The upper lobe along with the tumour was then removed (Figure 6). An intercostal drainage tube was inserted, and the patient was monitored in the intensive care unit for two days, before being transferred to the ward. His recovery was uneventful, and he was discharged seven days postoperatively. Histological examination revealed a complete excision with adequate margins of clearance. No evidence of lymphatic spread was observed. The patient was referred for adjuvant chemotherapy and received three cycles of chemotherapy. The patient will be followed up with CECT annually for the next five years.

## Discussion

Pulmonary resection in patients with colorectal cancer and lung metastasis has survival benefits [4-8]. DFI, number of lung lesions, and pre-thoracotomy CEA levels are considered important prognostic factors in patients undergoing lung resection for colorectal cancer with pulmonary metastasis [6,7].

DFI is defined as the time interval between colonic resection and lung resection. The median DFI described in the literature ranges from 18 to 37.5 months with the longest DFI being 260 months [4-7]. In the present case, the DFI was 240 months, which is a rare presentation. Studies have shown that the longer the DFI, the better the prognosis [7]; however, there are controversies [6].

Patients with normal pre-thoracotomy CEA levels are considered to have a better prognosis than those with elevated levels [5-7]. In this case, the patient had normal pre-thoracotomy CEA levels, which increased his survival benefits.

It is recommended that after performing a colonoscopy in the first year following curative surgery, the next colonoscopies should be performed at three years and five years postoperatively. Subsequent colonoscopies should be performed at five-year intervals [9]. The NCCN recommends CEA levels only up to five years after the primary resection. In our case, distant lung recurrence occurred 20 years after normal colonoscopy and in a patient with normal CEA levels. Notably, there are exceptions where patients present beyond the recommendations of guidelines.

## Conclusions

There are rare presentations of distant lung recurrence following curative surgery for rectal cancer, even after years. This should be considered during the follow-up management of patients with rectal cancer. Pulmonary resection in colorectal cancer with lung metastasis has survival benefits for the patient. DFI, number of lung lesions, and the pre-thoracotomy CEA levels are important prognostic factors.
